# A Robust Framework for Coffee Bean Package Label Recognition: Integrating Image Enhancement with Vision–Language OCR Models

**DOI:** 10.3390/s25206484

**Published:** 2025-10-20

**Authors:** Thi-Thu-Huong Le, Yeonjeong Hwang, Ahmada Yusril Kadiptya, JunYoung Son, Howon Kim

**Affiliations:** 1 Blockchain Platform Research Center, Pusan National University, Busan 609735, Republic of Korea; 2 School of Computer Science and Engineering, Pusan National University, Busan 609735, Republic of Korea; yeonjeong@islab.re.kr (Y.H.); yusril@pusan.ac.kr (A.Y.K.); jysonpaperinfo@gmail.com (J.S.)

**Keywords:** coffee bean package, label detection, image enhancement, image-to-text, instruction LLM, OCR, Qwen-VL, vision large model

## Abstract

Text recognition on coffee bean package labels is of great importance for product tracking and brand verification, but it poses a challenge due to variations in image quality, packaging materials, and environmental conditions. In this paper, we propose a pipeline that combines several image enhancement techniques and is followed by an Optical Character Recognition (OCR) model based on vision–language (VL) Qwen VL variants, conditioned by structured prompts. To facilitate the evaluation, we construct a coffee bean package image set containing two subsets, namely low-resolution (LRCB) and high-resolution coffee bean image sets (HRCB), enclosing multiple real-world challenges. These cases involve various packaging types (bottles and bags), label sides (front and back), rotation, and different illumination. To address the image quality problem, we design a dedicated preprocessing pipeline for package label situations. We develop and evaluate four Qwen-VL OCR variants with prompt engineering, which are compared against four baselines: DocTR, PaddleOCR, EasyOCR, and Tesseract. Extensive comparison using various metrics, including the Levenshtein distance, Cosine similarity, Jaccard index, Exact Match, BLEU score, and ROUGE scores (ROUGE-1, ROUGE-2, and ROUGE-L), proves significant improvements upon the baselines. In addition, the public POIE dataset validation test proves how well the framework can generalize, thus demonstrating its practicality and reliability for label recognition.

## 1. Introduction

Text reading of product package labels is a challenging task for many industrial and commercial applications, such as automatic inventory systems, product decoding, and supply chain tracking [1]. Correct label identification is also very important for brand identity checks, origin certification, and quality regulation compliance in the coffee domain  [2]. However, text extraction from coffee bean packages poses several challenges due to differently materialized packages (e.g., bottles and bags), differences in the position of the label (front and back), substantial variations in resolution and illumination, and geometric distortions, as well as typical image degradation factors, such as blur or noise [1].

Recent vision–language models (VLMs) have a proven state-of-the-art performance across multiple Optical Character Recognition (OCR) tasks, from general-purpose benchmarks [3] to specialized consumer product applications [4,5]. Despite these advancements, classic OCR systems often suffer from reduced robustness under tough, uncontrolled settings [6]. Coffee package labels exhibit these issues, with curved text on cylindrical surfaces, beautiful multilingual fonts, and sophisticated textured backdrops that set them apart from the more general areas covered by existing benchmarks. Furthermore, the lack of high-quality domain-specific repositories that reflect the real-world variance in coffee packaging has hampered the advancement and assessment of specialized OCR systems for this vertical [7,8]. OCEAN [3] evaluates OCR capabilities across many domains, but no dedicated datasets or pipelines are available for coffee package label extraction in industrial environments.

To address these issues, we introduce an end-to-end coffee bean package label recognition framework by fusing advanced image enhancement methods and structured prompt-based OCR models. Our solution is to create large-scale datasets including low- and high-resolution images, as well as a variety of back and front labels, multiple rotation angles, different illuminations, various packaging types, and varying levels of blur/distortion. We propose a preprocessing pipeline specifically tailored to improving image clarity, sharpness, and visual quality in general, consequently enabling more accurate text extraction.

Our approach uses four different versions of Qwen-VL OCR models with modified prompt-based instructions designed for coffee bean package label detection: Qwen2-VL-OCR-2B-Instruct [9], Nanonets-OCR-s [10], visionOCR-3B-061125 [11], and RolmOCR [12]. All models are trained on the Qwen/Qwen2-VL-2B-Instruct  [13,14] architecture and focus on product-specific label recognition tasks.

To perform a comprehensive evaluation, we compare the models to two widely used OCR baselines (Tesseract [15], EasyOCR  [16], DocTR (Document Text Recognition) [17], and PaddleOCR [18]), using multiple evaluation metrics including the Levenshtein distance  [19], Cosine similarity [20], Jaccard index [21,22], Exact Match [23], BLEU score [24], and ROUGE-N scores (ROUGE-1, ROUGE-2, and ROUGE-L [25]). We further verify our framework’s generalization ability with the public POIE dataset [26], showing that it performs impressively compared to various product types.

The main contributions of this paper are

- **Domain-specific datasets:** We introduce LRCB (Low-Resolution Coffee Bean) and HRCB (High-Resolution Coffee Bean), two new public datasets for coffee package label OCR. These datasets systematically capture real-world variations, including multiple resolutions, packaging types, label orientations, rotation angles, lighting conditions, and image quality degradations. To our knowledge, these are the first publicly available datasets specifically designed for coffee packaging OCR.
- **Multi-version preprocessing pipeline:** We develop a preprocessing module that generates three enhanced versions of each input image, optimized for the unique challenges of packaging materials (curved surfaces, reflections, variable lighting). This engineering solution improves the robustness in adverse real-world conditions without requiring model retraining.
- **Systematic evaluation of adapted VLMs:** We adapt four existing vision–language models through prompt tuning (CB-OCR-Qwen2-VL, CB-OCR-Nanonets, CB-OCR-RolmOCR, and CB-OCR-visionOCR-3B) and provide comprehensive comparisons against established OCR baselines (EasyOCR, Tesseract, DocTR, and PaddleOCR). This systematic benchmark provides practitioners with empirical guidance on model selection for packaging OCR tasks.
- **Comprehensive evaluation and generalization study:** We establish a thorough evaluation framework using multiple accuracy and similarity metrics. Additionally, we validate our pipeline on the public POIE dataset to demonstrate cross-domain generalization, showing that our preprocessing and adaptation strategies extend beyond coffee packaging to related document understanding tasks.

The remainder of this paper is organized as follows: In Section 2, we present the related work that has been carried out in OCR and text recognition for package imagery. The configurations of dataset construction, image preprocessing, and the OCR Qwen model with prompt-engineering models are presented in Section 3. The experimental results and performance analysis are given in Section 4. The paper concludes and some future directions of this research are discussed in Section 5.

## 2. Related Work

Text detection and recognition have been widely explored in the context of computer vision and document analysis. However, there continues to be a lack of enduring solutions among product package labels, particularly as relates to labels for coffee bean packaging. Such scarcity is mainly because of the difficulties posed by varying packaging materials, fluctuating image quality, strong blurring effects, and challenging real-world distortions. This section provides a detailed review of the related work in three important aspects: text detection and OCR approaches for product packages, prompt-enhanced vision–language models, and image enhancement methods in OCR. Our work extends these foundations by working with the most sophisticated image enhancement pipeline and applying it to VLM-OCR while guided by structured prompts on the coffee bean package label.

### 2.1. Text Detection and OCR on Product Packages

Text detection and recognition in product packaging scenarios have attracted much research interest because of their wide applications, such as automated checkout systems, inventory management, and brand authentication [6,27]. Tesseract [15] is widely used as it is open-source and performs well on document-like tasks, where all typescripts have the same layout and formatting. Other frameworks combine multilingual capabilities, such as EasyOCR [16], with easier setup methods, and have shown a better performance on curved and rotated text than that of traditional OCR systems.

Advanced traditional OCR systems have been developed to overcome the deficiencies of their ancestors. The proposed pre-trained model is compared with the state-of-the-art toolkit DocTR [17], which is an end-to-end trainable framework for document analysis comprising differentiable text detection and recognition modules, and achieves a more competitive performance. This architecture merges state-of-the-art convolutional structures and attention mechanisms together in order to deal effectively with difficult document layouts and different text orientations. PaddleOCR [18] is an end-to-end OCR production toolkit from Baidu and supports 80+ languages around the world. It also offers text detection models such as DB (Differentiable Binarization) with state-of-the-art accuracy and recognition models like CRNN. PaddleOCR particularly performs well on diverse text, including curved text, oriented text, and dense text, which widely exist in natural scenes and product packaging.

However, even with these improvements, traditional OCR models (e.g., DocTR and PaddleOCR) often suffer from significant performance degradation when subjected to real-world packaging scenarios. These include variable lighting conditions, perspective distortion, partial occlusions, reflections on surfaces, and cluttered background elements, all degrading the accuracy of text detection [28,29]. Though DocTR performs well on structured text documents, it has a suboptimal performance in the context of packaging images with an irregular text layout, curved surfaces, and material-dependent optical characteristics. At the same time, though PaddleOCR includes some advanced algorithms for text detection like others, it does not perform well when dealing with product packaging due to non-uniform illumination, surface reflections, complex graphics, and a lack of specialization in understanding.

Modern scene text recognition methods couple complex text localization algorithms with sequence-based recognition modules [30,31], which perform well on popular natural scene benchmarks. However, the image-based product packaging scenario poses unique challenges that are not common in scene text scenarios, including various material types for printing, deformation due to the label’s shape, differences in rotation, and optical properties specific to a material that have not been well handled by generic methods [32,33]. Even with advanced systems such as DocTR and PaddleOCR that involve deep learning methods, specialized adaptations to the specific features of materials used to package coffee beans (e.g., glossy surfaces, curved geometry, and different position layout patterns) are not possible. Methods that are specifically designed for packaging-related contexts, particularly for complex materials such as coffee bean packages with their distinctive surface properties and labeling arrangements, also remain an unmarked area in the literature [34].

### 2.2. Vision–Language Models and Prompt-Engineering Approaches

VLMs have achieved great success in multimodal learning, which has become a new standard for the understanding of multimodal tasks [35,36]. Prominent models like BLIP-2 [37], OFA [38], and Qwen-VL [14] have achieved a state-of-the-art performance in various cross-modal tasks, e.g., image captioning, visual question answering, and instruction-based interpretation.

Prompt-based fine-tuning methods have shown an effective strategy of increasing the model’s generalizability and adaptability to task-specific scenarios [39,40]. Carefully designed prompts allow the model to focus on selected regions and elements of text in an otherwise cluttered or visually complex context. Although these approaches have reported promising results in the field of natural image interpretation and document-oriented text harvesting tasks [41,42], few studies have focused on prompt-engineering strategies in domain-specific scenarios, especially with respect to product package label recognition. This omission becomes particularly obvious in more complex scenarios where the rotation angle varies, there are different lighting conditions, and the package edges are not regular. We fill this void by introducing and fine-tuning prompt-engineering techniques tailored to OCR in coffee bean package label settings.

### 2.3. Image Enhancement and Preprocessing for OCR

Image preprocessing is necessary for obtaining high recognition rates in OCR, especially when dealing with degraded or low-quality input imagery [43]. The traditional methods, such as histogram equalization, contrast enhancement, noise reduction, and mathematical morphology operators, were also used to enhance the legibility of text [44,45]. More advanced deep-learning-based restoration methods, including image super-resolution and deblurring, have shown potential in recovering fine details of text from challenging cases with severe degradation in the images [46,47,48,49].

Research-specific methods are applied in text-in-image to overcome problems like motion blur, poor contrast environment, non-uniform illumination, and background noise to achieve successful recognition rates. But the number of works that systematically concentrate on practical packing situations is relatively few. This gap is crucial because hard factors like reflective texture properties, non-uniform lighting distributions, motion blur effects, and material-dependent optical distortions can negatively affect the recognition performance [34,50]. The proposed architecture includes a specially designed image enhancement module to capture such packaging-induced lighting variations and, as a result, to improve robustness and accuracy in the recognition of coffee bean package labels.

### 2.4. Research Gap and Motivation

Although remarkable progress has been made in OCR techniques, VLMs, and image preprocessing methods, there remain limited domain-adapted frameworks for coffee bean package label recognition. Existing works on product label OCR (e.g., POIE, DocBank datasets) primarily focus on broad categories of consumer goods or documents, but they do not fully address the unique optical and design properties of coffee packaging. These include glossy or metallic surfaces, small multilingual fonts, non-uniform layouts, and curved geometries.

Building on this foundation, we propose an elaborate framework that integrates VLMs with a tailored preprocessing pipeline and a structured prompting strategy, offering a domain-specialized solution for robust coffee bean package label recognition. The proposed integrated system is designed for the unique characteristics of coffee packaging, delivering high accuracy and robustness across diverse real-world operational scenarios.

## 3. Methodology

**Figure 1:**
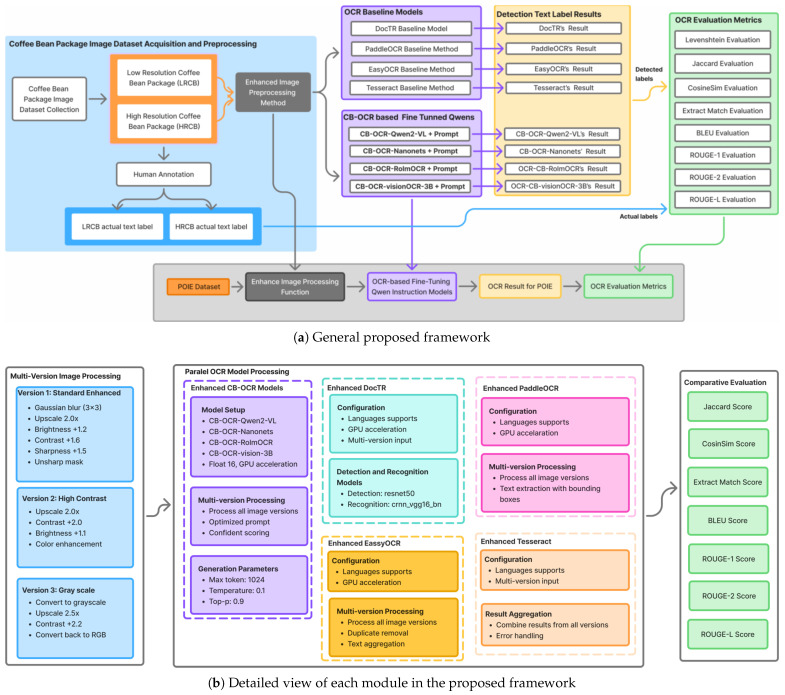
The overall framework of our proposed OCR coffee bean package label recognition system, including dataset acquisition, preprocessing, OCR model comparisons, and evaluation metrics.

Our designed framework for robust text label recognition of coffee bean package images is motivated by the challenges in accurately recognizing text under varying imaging conditions, resolutions, and packaging designs. Existing OCR approaches often fail to generalize across low- and high-resolution images or to effectively combine insights from multiple OCR models. To address these limitations, we propose a comprehensive framework that ensures both a high recognition accuracy and adaptability to domain-specific text layouts. The framework consists of five main components (see Figure 1): dataset building and acquisition, multi-version image preprocessing, parallel OCR model processing, result combination, and global evaluation metrics.

As illustrated in Figure 1a, the framework begins by constructing two datasets: Low-Resolution Coffee Bean (LRCB) and High-Resolution Coffee Bean (HRCB). Human annotation is employed to generate reliable ground truth labels, ensuring high-quality supervision for model training. To further test generalization, we incorporate the public POIE dataset, which includes varied document types and imaging conditions. The systematic image collection and annotation process addresses the scarcity of high-quality, domain-specific labeled data, a key limitation in previous OCR research for industrial packaging.

To capture a holistic understanding of the model performance across different aspects of text recognition, we employ eight evaluation metrics. The Levenshtein distance measures the character-level accuracy by computing the minimum edit distance between predicted and ground truth labels. The Jaccard index evaluates the token-level overlap. Cosine similarity measures semantic similarity using TF-IDF vector representations. Exact Match quantifies the percentage of perfect matches between the predictions and ground truth. The BLEU score assesses n-gram precision. ROUGE-1, ROUGE-2, and ROUGE-L evaluate the unigram overlap, bigram overlap, and longest common subsequence, respectively, capturing both lexical and structural similarities.

The modular design of our framework allows for controlled dataset processing, advanced image enhancement, parallel OCR model execution, and thorough evaluation. Parallel processing bridges traditional OCR approaches with modern vision–language models, enabling an effective combination of complementary strengths. The need to enhance both the recognition robustness and processing efficiency in domain-specific applications motivated this approach. By explicitly addressing limitations in prior works, our framework offers practical guidance for selecting and adapting OCR paradigms in industrial coffee bean package label recognition.

### 3.1. Coffee Bean Package Image Collection and Dataset Construction

#### 3.1.1. Dataset Overview and Motivation

Coffee bean packaging OCR has its own characteristics, including the diversity of materials, typefaces, and real imaging environments. Unfortunately, current public datasets have limited diverse packaging types and viewpoints, as well as variations in image quality and scene conditions, which is not ideal for training robust OCR models to handle commercial coffee packaging in an inference task.

To partly address these contexts, we have created two datasets that can represent a variety of challenges in the wild for the label reading task in coffee bean packages: the Low-Resolution Coffee Bean (LRCB) dataset and the High-Resolution Coffee Bean (HRCB) dataset. These datasets include a variety of real-world and packing variations that commercial OCR systems must deal with.

#### 3.1.2. Dataset Specifications and Characteristics

The LRCB dataset contains 656 images with a resolution between 480×640 and 800×600 pixels and simulates them using widely used smartphone cameras for different scenarios. These images simulate the quality and resolution that can be encountered in non-industrial products, mobile scanning environments, and very low-end imagers. The HRCB dataset consists of 503 high-quality images with a resolution of 1920×1080 to 4000×3000 pixels taken using professional cameras and high-end mobile devices. This collection illustrates situations where high-end imaging equipment is used, such as professional product photography or scans of industrial machinery.

Both datasets contain many different packaging materials, which are produced in coffee branches. Types include glass bottles, clear and colored variations, with diverse label attachment methods, and plastic containers with stiff packaging, to which printed labels and adhesive are applied. In order to gain comprehensive coverage of the real-life scanning situation, the acquisition is performed from several points of view and directions. This also includes different viewing angles, such as front label, back label, side labels, and angled perspectives, as well as 0°, 90°, 180°, and 270°rotations, which simulate different handling conditions. Also, the distance fluctuates by featuring closeups to specific text areas, along with full-package shots.

To ensure the robustness of the algorithms under realistic conditions, the datasets intentionally include a variety of image quality degradations and environmental variations. These include blur effects, such as motion blur, defocus blur, and camera shake; lighting variations, including natural daylight, artificial indoor lighting, low-light nighttime conditions, and combinations of multiple light sources; complex backgrounds with varying amounts of clutter and contrast; and reflective surfaces producing highlights on glossy materials.

#### 3.1.3. Web Crawling Framework and Data Collection Methodology

These heterogeneous datasets of different qualities were generated using an in-house web crawling platform with Selenium WebDriver and BeautifulSoup. This dual approach of extracting data from JavaScript-heavy web pages and organizing the resulting code in a uniform structure also provides useful information to developers in a readable way while maintaining efficiency. The crawler is designed to collect product pages from the World Open Food Facts database [51], an extensive and open-source database that contains a detailed description of food products across the world. This data presents a collection of images of fronts and backs or only fronts captured in various real-world situations, which makes it very suitable for the coffee bean packaging dataset.

The architecture of the crawling system consists of several key components:

- Selenium WebDriver [52] automates the web browser and is necessary to load JavaScript-based content, which is a requirement for modern web applications making use of dynamic content loading and client-side rendering.
- Beautiful Soup is used for correct HTML parsing, the extraction of the DOM elements where product description and image links are provided, and handling non-standardized HTML and complex page structures.
- We undertake a basic quality check to ensure that images are of an acceptable resolution and quality before download: both retain dataset quality and reduce the amount of manual curation.

Our crawling algorithm is described in detail in Algorithm 1:
**Algorithm 1** Image acquisition and preprocessing of coffee bean packages.**Require:** 
Page starting point *s*, page end point *e*, base URL, request headers, save directory path**Ensure:** 
Image databanks with their metadata collected1:If the directory structure for saving images does not exist, create it2:Selenium Chrome WebDriver initialization (with optimized options)3:Logging and progress tracking facilities will be set up4:**for** each page number *p* between *s* and *e* **do**5:    BUILD URL of search with page number *p* AND coffee-specific filters6:    Open the URL with Selenium and let dynamic content load7:    Parse the fully rendered page source with Beautiful Soup8:    Obtain all product links from the current search result page9:    **for** each product link *ℓ* in the generated links **do**10:        Crawl the product page HTML using HTTP requests and headers11:        Parse the contents and key information from the HTML source code12:        **Front Image Processing:**13:           Locate front image container tags as per CSS selector(s)14:           Verify image URL and resolution requirements15:           Download a high-resolution front label image if possible16:        **Back Image Processing:**17:           Search for ingredient/back label image tags18:           Quality filter and resolution check19:           Retrieve back label image with ingredient content20:        Name the file according to the code and type of image, date, and time21:        Store images and their related metadata (resolution, source, etc.)22:        Log the progress and handle downloading errors generously23:    **end for**24:    Apply rate limiting to avoid server overloading25:**end for**26:Quit Selenium WebDriver and clean up the temporary data27:Create summary statistics of the dataset and a quality report

Following the initial collection phase, we undertook several quality assurance steps to preserve the completeness and utility of the dataset for OCR model training:

- Automated quality check through computer vision to reject images with low-quality issues that are too extreme, such as extreme blur, overexposure, or insufficient text;
- Perceptual hashing for near-duplicate image detection and removal, to increase dataset diversity and to avoid overfitting the model during training;
- Image conversion into the same format with quality at a standard level for operating the process faster and at the same time not losing what is important for an OCR task: image visual quality.

After collection and quality assurance of the data were performed automatically, extensive manual annotation was conducted to generate ground truth labels for text detection and recognition. This annotation process includes

- Human annotators mark all of the visible text regions in each image by providing the bounding box annotations, with an emphasis on challenging situations, like curved text, or when it curves over other text or lies against a complex background;
- Manual transcription of all identified text regions into textual form with high accuracy, taking care of the specific special characters and multilanguage textual and font styles widely used on coffee packaging;
- A multi-level (stages) annotation scheme supported through repeated cross-validations by many annotators for consistency and accuracy, with disagreements resolved in joint review sessions;
- Quality ground truth with high standards for OCR data struck through accurate main text content (as well as the international character set and symbols).

#### 3.1.4. Annotation Schema and Quality Control

To ensure a reliable ground truth for coffee bean package OCR, we designed a rigorous annotation workflow combining manual labeling and systematic quality checks. The annotation process was defined as follows:

- Annotation granularity. Bounding box annotation was performed at the word level, with each word in the image enclosed by a tight polygonal box. This level of annotation was chosen because it balances OCR training utility with annotation feasibility for multilingual, multi-font coffee packaging. Although our primary unit of annotation was the word, character-level transcription checks were conducted to verify the correctness of special characters, diacritics, and multilingual text (e.g., accented Latin scripts and Asian characters). Line- and paragraph-level annotations were not included, as packaging designs typically involve scattered and stylistically varied text elements.
- Transcription. Each annotated bounding box was manually transcribed with strict fidelity to the printed text, including punctuation marks, brand-specific typography, and multilingual characters. Annotators were instructed to retain the case sensitivity and spacing exactly as observed.
- Quality control. To validate annotation consistency, we adopted a multi-stage cross-check procedure: Redundant annotation: Each image was independently annotated by two annotators. Agreement metrics: Bounding box consistency was measured using the Intersection over Union (IoU) with a threshold of 0.75. Text transcription consistency was quantified using the F1 score at the character level. Resolution of disagreements: Cases with IoU <0.75 or F1 <0.9 were flagged for review. Discrepancies were resolved in joint review sessions involving a senior annotator.

#### 3.1.5. Dataset Statistics and Analysis

The datasets presented in the following sections offer a wide range of coverage of various coffee bean packaging, as shown in Table 1.

These neatly prepared datasets provide a strong foundation for training and testing of custom OCR frameworks that deal with coffee bean packaging recognition. They are diverse and fine enough for practical real-world use.

### 3.2. Image Preprocessing Pipeline

Our dedicated preprocessing pipeline addresses the specific difficulties in coffee bean package images with three parallel enhancement strategies. These methods focus on certain degradation patterns that often appear in product packaging situations:

- **Standard Enhanced**: Gaussian filtering is conducted on the noise in the original LR image; then, bicubic interpolation up to 2× is applied. Brightness and contrast are processed using adaptive histogram equalization and sharpening using an unsharp mask with hand-tuned parameters.
- The **High-Contrast** set of images is obtained through the application of CLAHE (Contrast Limited Adaptive Histogram Equalization) to improving the local contrast without overshooting. The color channels are normalized to equalize the color distribution, and selective contrast control is performed using local image statistics.
- **Grayscale-Based** casts the image into grayscale using a luminance-preserving method to reduce chromatic noise and enhance text–background contrast. Super-resolution is achieved with bicubic interpolation, using edge-preserving filters and aggressive contrast enhancement and restoring the RGB by duplicating the channels.

Due to large variations in image quality, packing materials, and shooting environments, we put forward an upgraded image preprocessing pipeline, which can remove the background noise and make characters more visible for OCR. This I=image preprocessing step, shown in Figure 1, is critical to achieving a robust text detection and recognition performance, especially for scenes with poor contrast, heavy blur, or uneven illumination in real scenarios.

The preprocessing framework exhaustively generates three enhanced versions of each input image, providing diverse methods to improve the text extraction performance. These versions are specifically designed to address common types of degradation observed in real-world coffee bean packaging images. Our preprocessing approach is described in pseudocode in Algorithm 2.
**Algorithm 2** CreateMultipleVersions: enhanced image preprocessing.1:**Input:** Image *I*2:**Output:** Preprocessed versions $V=\{V^{\left(1\right)},V^{\left(2\right)},V^{\left(3\right)}\}$3:$I_{\mathrm{base}}\leftarrow \mathrm{LoadImage}\left(I\right)\to \mathrm{RGB}$4:$I_{\mathrm{base}}\leftarrow \mathrm{ExifTranspose}\left(I_{\mathrm{base}}\right)$5:                  ▷ Version 1: Standard Enhanced Preprocessing6:$V^{\left(1\right)}\leftarrow \mathrm{PreprocessImageForOCR}\left(I\right)$7:                    ▷ Version 2: High Contrast and Brightness8:$V^{\left(2\right)}\leftarrow \mathrm{Resize}\left(I_{\mathrm{base}},\mathrm{factor}=2.0\right)$9:$V^{\left(2\right)}\leftarrow \mathrm{EnhanceContrast}\left(V^{\left(2\right)},\mathrm{factor}=2.0\right)$10:$V^{\left(2\right)}\leftarrow \mathrm{EnhanceBrightness}\left(V^{\left(2\right)},\mathrm{factor}=1.1\right)$11:                      ▷ Version 3: Grayscale High Contrast12:$V^{\left(3\right)}\leftarrow \mathrm{ToGrayscale}\left(I_{\mathrm{base}}\right)$13:$V^{\left(3\right)}\leftarrow \mathrm{Resize}\left(V^{\left(3\right)},\mathrm{factor}=2.5\right)$14:$V^{\left(3\right)}\leftarrow \mathrm{EnhanceContrast}\left(V^{\left(3\right)},\mathrm{factor}=2.2\right)$15:$V^{\left(3\right)}\leftarrow \mathrm{ToRGB}\left(V^{\left(3\right)}\right)$16:**return**$V=\{V^{\left(1\right)},V^{\left(2\right)},V^{\left(3\right)}\}$

Every input image is fed through a dedicated preprocessing pipeline made up of three transformation branches (see Figure 1b), which are designed to improve text visibility and structural coherence from various imaging conditions:

- **Standard Enhanced:** The branch uses a 3 × 3 Gaussian blur kernel, upsamples images 2.0×, brightens +1.2, contrast-enhances +1.6, and texture-sharpens by +1.5, applied with unsharp masking. This release should function as a standalone, generic enhancement pipeline, targeting denoising, deblurring, and normalization for human-readable, common product labeling.
- **High Contrast:** Upscale by 2.0× and increase fine-tuning contrast level by +2.0 and fine-tuning brightness by 1.1, and color-boost. This setting is specifically designed to boost the local contrast and enhance faint or partially obscured text, especially in reflective or low-contrast areas.
- **Grayscale:** This branch converts images into grayscale, scales up by 2.5×, and then applies contrast enhancement of +2.2, before converting back into RGB format. Unlike the vanilla variant, this variant aims to have clear edges in text and features fine contours on individual text. This will help easily define gradients for OCR models using 3-channel inputs, such that a single channel is enough to combine information from three RGB channels.

This multi-version preprocessing indeed increases the diversity of visual representations, which greatly promotes the robustness and flexibility of the OCR pipeline. Thus, our methods can efficiently choose or fuse the informative image variations so as to enhance the robustness and accuracy of text recognition for challenging high-resolution coffee packaging images.

### 3.3. CB-OCR: Qwen-Based Models for Text Label Recognition

The main algorithm proposed is described in Algorithm 3. The algorithm has several key features: (i) a unified model selection framework covering all four VL models; (ii) a dynamic model loading mechanism that automatically applies the selected model based on user choice; and (iii) a model-specific configuration, with fine-tuned parameters and structured prompts for each of the four models. Additionally, to the best of our knowledge, all models are evaluated under identical preprocessing and evaluation metrics, and the framework provides a configurable setup that allows new models or modifications to existing models to be easily incorporated.
**Algorithm 3** Process_Model: Multi-variant OCR with best-candidate selection.1:**Input:** Image *V*, prompt *p*, VL model VLM, processor Processor, model type $M_{\mathrm{type}}$2:**Output:** OCR result $T_{\mathrm{output}}$3:image_versions←CreateMultipleVersions(V)4:best_result←"", best_confidence←05:**for all** img∈image_versions **do**6:    Format inputs according to $M_{\mathrm{type}}$ using Processor7:    generated←VLM.generate(inputs,max_new_tokens=1024,temperature=0.1--0.2)8:    decoded←Processor.batch_decode(generated)9:    cleaned←RemoveSpecialTokens(decoded)10:    **if** len(cleaned)>best_confidence∧len(cleaned)>5 **then**11:        best_result←cleaned12:        best_confidence←len(cleaned)13:    **end if**14:**end for**15:$T_{\mathrm{output}}\leftarrow best\_result$16:**return** $T_{\mathrm{output}}$

After OCR, all results are passed through a complex result aggregation component tailored to discard duplicated predictions among multiple image versions, merge related text fragments without breaking semantic consistency, correct errors via majority voting, and select coherent final decisions confidently. This consolidation ensures consistency across multiple processing versions and model outputs, producing accurate and robust text extraction results.

We build domain-specific textual prompts using the pre-trained Qwen-VL as the backbone—for example,

“Extract all visible text from this coffee package image. Be thorough and accurate.”

This prompt encourages the model to focus on label text under challenging conditions such as blur, glare, small multilingual fonts, and non-standard layouts. The prompts were carefully crafted and internally validated; while a systematic comparison of zero-shot, few-shot, or general wording prompts was not conducted, preliminary experiments showed that domain-specific prompts consistently achieved the best recognition accuracy and robustness. A comprehensive prompt sensitivity study is planned as future work.

The CB-OCR variants used are summarized as CB-OCR-Qwen2-VL + Prompt (Model 1), CB-OCR-Nanonets + Prompt (Model 2), CB-OCR-RolmOCR + Prompt (Model 3), and CB-OCR-visionOCR-3B + Prompt (Model 4). These models are fine-tuned on our proprietary LRCB and HRCB datasets for domain-specific adaptation to diverse packaging materials, label positions, imaging conditions, and rotations.

To fully utilize Qwen-VL for coffee bean package label text detection, we propose a prompt-engineering strategy. This involves crafting guided instructions that direct the model to extract text from regions with round surfaces, reflective materials, and varying viewing angles. Prompt templates are organized as follows:

“You are an expert OCR system specialized in reading text from coffee bean package labels. Please extract all visible text from this image, including brand names, product descriptions, ingredients, and any other textual information.”

The prompts are adaptively produced based on image properties discovered during preprocessing, with additional guidance for improving detection on blurry or low-contrast images. In cases with multiple text regions, the prompts provide instructions for systematic region-wise scanning and contour extraction. This domain-specialized design of prompts is critical to the robustness and accuracy of our CB-OCR models.

Finally, to explicitly clarify prompt validation, the structured prompts used in the CB-OCR models were specifically designed and internally validated for coffee bean package labels; while a full comparison across alternative prompt designs was not performed, these domain-specific prompts consistently provided the best recognition accuracy and robustness.

At the same time, we use enhanced versions of standard OCR engines:

- **DocTR:** Structured with language support, GPU acceleration, and the capability to process multiple versions of input and implementing a detection mechanism using ResNet50, while the recognition architecture is based on CRNN.
- **PaddleOCR:** Features support for multiple languages and GPU acceleration, with all image variants processed where text is extracted and bounding boxes detected.
- **EasyOCR:** This provides support for multiple languages with GPU acceleration, processes all image versions well, and features means of duplicate removal and text organization.
- **Tesseract:** Composed of language support and multi-version input processing, it includes result aggregation to combine results from all versions and error handler abilities.

### 3.4. Evaluation Metrics and Performance Assessment

For a robust assessment of OCR quality, we use eight complementary metrics that preserve all three dimensions of recognition quality in terms of characters, words, and semantics, as illustrated by Algorithm 4. These metrics enable an all-round evaluation of syntactic correctness, semantic similarity, and linguistic fidelity under different scenarios of coffee bean package labels. For an additional assessment of the generalization ability, we apply our OCR pipeline on the POIE dataset [26], a published dataset for unlabeled image retrieval, with a similar preprocessing and prompt-based OCR approach to that shown in Figure 1.
**Algorithm 4** ComputeMetrics: Comprehensive evaluation of OCR results.1:**Input:** Predicted text $T_{P}$, ground truth text $T_{G}$2:**Output:** Metric set $M=\left\{\mathrm{Lev},\mathrm{Cos}\mathrm{Sim},\mathrm{Jaccard},\mathrm{EM},\mathrm{BLEU},\mathrm{ROUGE}-1,\mathrm{ROUGE}-2,\mathrm{ROUGE}-L\right\}$3:**if** $T_{P}=\emptyset$ **or** $T_{G}=\emptyset$ **then**4:    **return** all metrics = 05:**end if**6:Compute LevenshteinRatio$\left(T_{P},T_{G}\right)$7:Compute TF-IDF-based CosineSimilarity$\left(T_{P},T_{G}\right)$8:Compute JaccardIndex$\left(T_{P},T_{G}\right)$9:Compute ExactMatch$\left(T_{P},T_{G}\right)$10:Compute BLEU$\left(T_{P},T_{G}\right)$11:Compute ROUGE-1, ROUGE-2, ROUGE-L$\left(T_{P},T_{G}\right)$12:M←{Lev,CosSim,Jaccard,EM,BLEU,ROUGE-1,ROUGE-2,ROUGE-L}13:**return** *M*

**Levenshtein distance (Lev):** The Levenshtein distance measures the minimum number of single-character edits required to transform the predicted text *s* into the ground truth *t*:

(1) $$d\left(s,t\right)=\min\left\{\begin{array}{cc} d(s_{1..i-1},t_{1..j})+1 & \left(\mathrm{deletion}\right)\\ d(s_{1..i},t_{1..j-1})+1 & \left(\mathrm{insertion}\right)\\ d(s_{1..i-1},t_{1..j-1})+\cos t & \left(\mathrm{substitution}\right) \end{array}\right.$$

where cost=0 if $s_{i}=t_{j}$; otherwise, it is 1.

To interpret this distance in terms of similarity, we define the **Levenshtein similarity** as

(2) $$\mathrm{LevSim}\left(s,t\right)=1-\frac{d(s,t)}{\max(|s|,|t|)},$$

where |s| and |t| are the lengths of *s* and *t*, respectively. This normalization converts the distance into a similarity score between 0 and 1, where higher values indicate better alignment between the predicted and ground truth texts.

**Cosine similarity (CosSim):** Cosine similarity evaluates semantic closeness by comparing TF-IDF vector representations:

(3) $$\mathrm{CosineSim}\left(A,B\right)=\frac{A\cdot B}{∥A∥\;∥B∥}$$

where *A* and *B* are TF-IDF vectors, · denotes the dot product, and ∥·∥ the Euclidean norm. Values closer to 1 indicate stronger semantic alignment.

**Jaccard index (Jaccard):** The Jaccard index measures the token-level overlap between predicted (*P*) and ground truth (*G*) word sets:

(4) $$\mathrm{Jaccard}\left(P,G\right)=\frac{\left|P\cap G\right|}{\left|P\cup G\right|}$$

A higher score reflects greater similarity in the recognized content. –> it is fine

**Exact Match (EM):** The EM metric assigns a binary score:

(5) $$\mathrm{EM}\left(P,G\right)=\left\{\begin{array}{cc} 1, & P=G\\ 0, & P\neq G \end{array}\right.$$

Both the predicted text *P* and ground truth *G* are normalized to avoid trivial format discrepancies before calculating the Exact Match (EM). This normalization includes lowercasing, trimming (leading and terminating spaces are removed), unifying whitespaces, removing non-significant punctuation, and harmonizing the spacing between numbers and units by hand (e.g., both ‘100 g’ and ‘100 g’ will be set to ‘100 g’). These steps ensure that the EM metric correctly captures substantive correctness, as opposed to spurious differences due to OCR layout.

**BLEU score (BLEU):** BLEU measures the n-gram precision between predicted and ground truth sequences, incorporating a brevity penalty:

(6) $$\mathrm{BLEU}=\mathrm{BP}\cdot \exp\left(\sum_{n=1}^{N}w_{n}\log p_{n}\right)$$

where $p_{n}$ is the precision for n-grams, $w_{n}$ are weights, and BP is the brevity penalty. BLEU is sensitive to word order and fluency.

**ROUGE-N (ROUGE-1 and ROUGE-2):** ROUGE-N evaluates n-gram recall. For ROUGE-1 and ROUGE-2, unigram and bigram matches are computed:

(7) $$\mathrm{ROUGE}-N=\frac{\sum_{n-\mathrm{gram}\in \mathrm{Ref}}\min\left({\mathrm{Count}}_{\mathrm{pred}},{\mathrm{Count}}_{\mathrm{ref}}\right)}{\sum_{n-\mathrm{gram}\in \mathrm{Ref}}{\mathrm{Count}}_{\mathrm{ref}}}$$

This metric emphasizes coverage of reference content.

**ROUGE-L:** ROUGE-L uses the longest common subsequence (LCS) to capture fluency and sentence-level coherence:

(8) $$\mathrm{ROUGE}-L=\frac{\mathrm{LCS}(P,G)}{\left|G\right|}$$

where LCS(P,G) is the length of the longest common subsequence between the predicted and ground truth text.

Together, these eight metrics provide a holistic view of OCR quality, spanning from exact correctness to semantic similarity and linguistic recall.

## 4. Experiments and Discussion

All our experiments were performed in Python 3 on a high-performance computer system with an Intel(R) Core(TM) i7-10700K CPU @ 3.80 GHz, 64 GB of RAM, and an NVIDIA H100 GPU (Driver Version: 525.105.17; CUDA Version: 12.0). This setup enabled an efficient large-scale evaluation of our CB-OCR models on multiple datasets. The four CB-OCR models’ hyper-parameters are presented in Table 2.

For fair comparison, all VLM-based models were run with a batch size of 1 and the input images resized to a maximum of 1024 pixels on the longer edge, utilizing the GPU. We present in this section the results for model performance, quantitative evaluations across accuracy and similarity measures, inference speed comparison, and qualitative rendering of label detection.

### 4.1. Enhanced Image Processing

**Figure 2:**
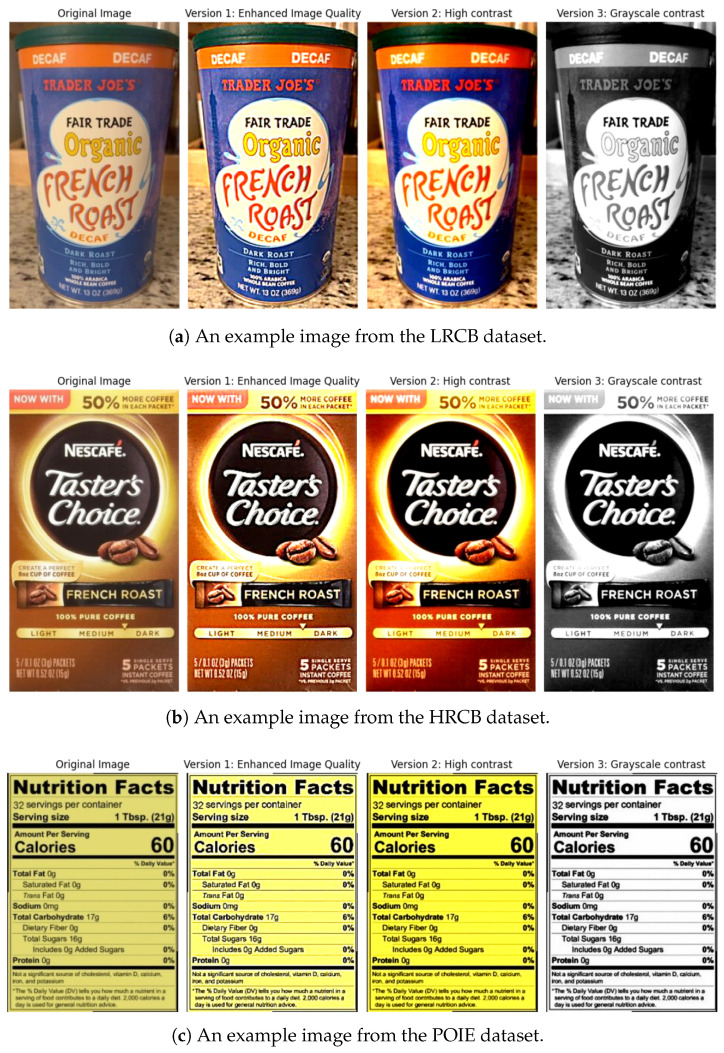
Enhanced image preprocessing input data for CB-OCR models: (**a**) LRCB dataset, (**b**) HRCB dataset, and (**c**) POIE dataset. Each image shows the original input alongside three processed versions: Version 1 (enhanced image quality), Version 2 (high contrast), and Version 3 (grayscale contrast).

The improved image preprocessing method presented in Algorithm 2 should ultimately enhance text visibility and robustness to popular conditions of packaging and imaging. Its use is illustrated in Figure 2, as applied to the LRCB, HRCB, and POIE datasets.

In the LRCB dataset (Figure 2a), our preprocessing reveals text, including the brand and roast, from Trader Joe’s packaging that is not visible due to poor lighting and reflection. On the HRCB dataset (Figure 2b), it succeeded in achieving a large improvement on Nescafé packaging, where strong color differences and glossiness surfaces create untexturized regions. For the nutrition fact/ingredient table POIE coding (Figure 2c), the model preserves the fine-print readability quality and can slightly tone the binarization for better metrics.

This systematic preprocessing will guarantee that all the downstream OCR models will receive nice and varied, yet reliable, high-quality inputs, for better text detection and recognition on their end. It also makes the OCR models less sensitive to lighting, print quality, and packing material, which is crucial for robust analysis in a real-world retail store.

### 4.2. Performance Evaluation on Coffee Bean Product Image Datasets

We further demonstrated the superior performance of our proposed CB-OCR models via a comprehensive set of experimental evaluations against baseline OCR methods on three representative datasets: LRCB, HRCB, and the publicly available POIEx dataset in extensive settings. All results were generated on processed image versions obtained from our preprocessing pipeline (Algorithm 2) to make a fair comparison between all methods.

To evaluate OCR performance, we used eight evaluation metrics: the Levenshtein similarity, Jaccard index, Cosine similarity, Exact Match, BLEU, ROUGE-1, ROUGE-2, and ROUGE-L. Higher similarity scores and metric values are better for OCR. A global summary of the average competitor approaches per metric is shown in Table 3.

The results show that there are different speed/efficiency performances for different datasets and evaluation metrics. On the LRCB dataset, most metrics are surpassed by CB-OCR-RolmOCR, with notably high scores for Cosine similarity (0.8099), Exact Match (0.7852), and ROUGE-1 (0.7302). The results show that there is very strong semantic image comprehension and a retained text structure in low-resolution coffee bean packaging images.

In the HRCB dataset, both CB-OCR-RolmOCR and CB-OCR-Qwen2-VL exhibit competitive memory usage as well. Although CB-OCR-Qwen2-VL achieves the best Levenshtein similarity (0.2320), the structural metrics are in favor of CB-OCR-RolmOCR, with a Jaccard index of 0.3641, an Exact Match of 0.5280, and better ROUGE scores. This indicates the complement in expertise between the models in handling high-resolution images.

The largest discrepancy in performance is observed on the POIE dataset. CB-OCR-RolmOCR demonstrates significant effectiveness in all metrics, including the superior performance in the Levenshtein similarity (0.3700), Jaccard index(0.4148), and Exact Match (0.7525), as well as various ROUGE scores. Notably, it achieves a ROUGE-1 score of 0.9136, which means a significant performance for structured product information extraction, such as nutrition facts and ingredient lists. On the other hand, CB-OCR-Qwen2-VL has a similar Cosine similarity (0.6640), indicating that the semantic vector representation is good.

A number of important insights for the realistic deployment of CB-OCR models are provided by our in-depth analysis. No single CB-OCR variant outperforms others in all datasets and evaluation measures; hence, task-specific model selection is crucial. CB-OCR-RolmOCR consistently outperforms competitors in structured text extraction and semantic integration, doing very well on the difficult POIE task containing challenging tabular information.

For use cases that favor semantic understanding and structured text extraction, CB-OCR-RolmOCR demonstrates an outstanding performance, having obtained top scores in the majority of the similarity measurements. It is thus the favored choice for parsing complex tabular data, nutritional information, and other well-structured product content that often appears on coffee packaging.

CB-OCR-Qwen2-VL demonstrates an impressive performance in semantic vector representation (Cosine similarity), which can also deliver competitive performances under several common application situations, making it a good candidate for applications that need a good strength of semantic matching.

The findings provide practical recommendations on selecting the optimal compositions of the CB-OCR models for particular application scenarios, dataset statistics, and performance necessities. Finally, it increases the applicability of automatic coffee product information extraction systems to real-world usage settings.

### 4.3. Illustrating CB-OCR Models for Label Detection Performance

**Figure 3:**
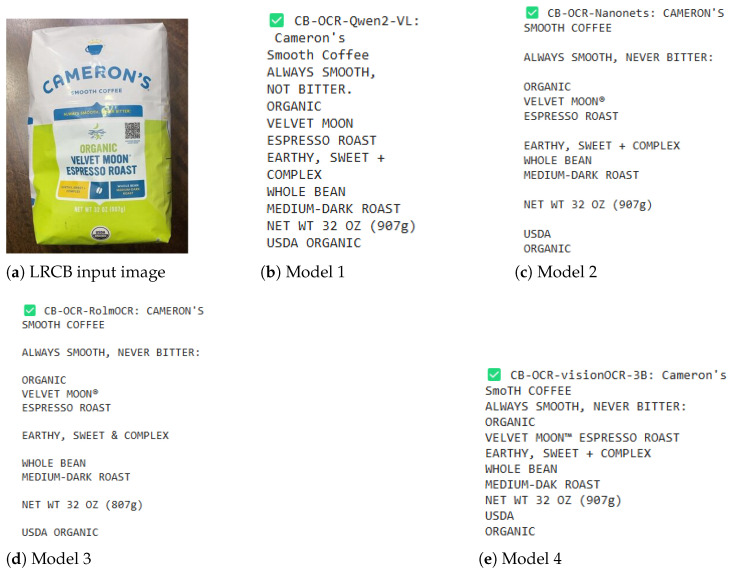
Label detection performance comparison for CB-OCR models on an LRCB dataset example. (**a**) Original coffee bean product label. (**b**) CB-OCR-Qwen2-VL. (**c**) CB-OCR-Nanonets. (**d**) CB-OCR-RolmOCR. (**e**) CB-OCR-visionOCR-3B.

**Figure 4:**
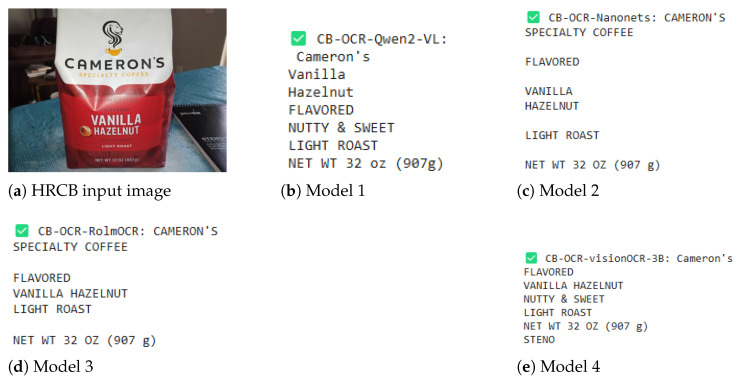
Label detection performance comparison for CB-OCR models on an HRCB dataset example. The figure displays (**a**) the original label image, followed by OCR extraction results from (**b**) CB-OCR-Qwen2-VL, (**c**) CB-OCR-Nanonets, (**d**) CB-OCR-RolmOCR, and (**e**) CB-OCR-visionOCR-3B models.

**Figure 5:**
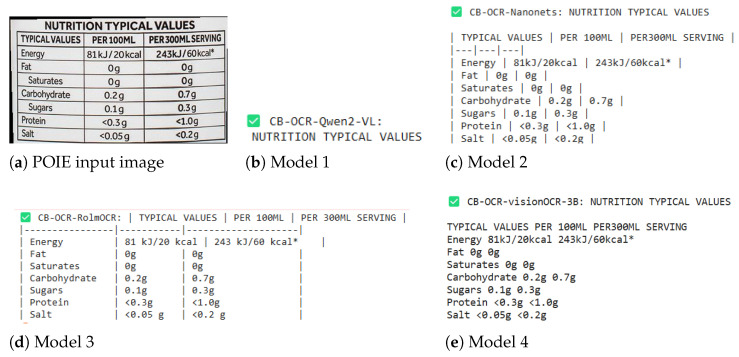
Label detection performance comparison for CB-OCR models on a POIE dataset example. The figure illustrates (**a**) the original nutrition fact label and the corresponding OCR results from the (**b**) CB-OCR-Qwen2-VL, (**c**) CB-OCR-Nanonets, (**d**) CB-OCR-RolmOCR, and (**e**) CB-OCR-visionOCR-3B models.

In addition to the quantitative results, for a better understanding of what can be achieved with the proposed CB-OCR models in real practice for label detection, we provide several complete visual examples and text extraction comparisons on three different datasets: LRCB, HRCB, and POIE. These examples serve to showcase models’ behavior with relation to different types of labels, as well as various complexities, and are shown in Figure 3, Figure 4 and Figure 5.

The example from the LRCB dataset—see Figure 3—demonstrates a product label for coffee beans consisting of detailed textual specification types spread across multiple lines, such as roast type category, details on flavor notes, and various certifications. Of the evaluated models, CB-OCR-Qwen2-VL is noteworthy in its outstanding performance by keeping almost all semantic elements of the source label and original line structure; thus, it gives quite a faithful textual description for the labeled image. For CB-OCR-Nanonets and CB-OCR-RolmOCR, they do preserve all of the prominent information components, however, with some variants in the line formatting and sometimes missing some text. On the other hand, although CB-OCR-visionOCR-3B has generally a good performance, it introduces some random differences for line breaks and text spaces.

As an example from the HRCB dataset (Figure 4), a simple but elaborate text block mentions flavor profiles (vanilla, hazelnut) and roast level information. CB-OCR-Qwen2-VL consistently maintains better text fidelity with improved readability and accurately recognizes the key terms, compared to preserving the layout structure. CB-OCR-Nanonets and CB-OCR-RolmOCR seem the best options, but they mostly have moderate effects, as well as introducing unwanted line breaks or awkward definitions between words. CB-OCR-visionOCR-3B is able to keep the important textual content, but it has some slight positional aberrations and extra spacing issues.

Figure 5 illustrates the OCR outputs from all CB-OCR model variants on a nutrition fact label from the POIE dataset, which is characterized by structured tabular formatting and small-font numeric fields. This example is used to assess how well each model preserves both tabular alignment and semantic fidelity in the extracted text.

- CB-OCR-Qwen2-VL (Model 1) demonstrates concise recognition, summarizing key textual information but occasionally omitting less prominent entries, such as secondary numeric units.
- CB-OCR-Nanonets (Model 2) exhibits strong structural retention, preserving most table delimiters and column alignment, which enhances readability for downstream structured parsing.
- CB-OCR-RolmOCR (Model 3) produces the most faithful reproduction of both textual and numeric information, effectively maintaining cell structure and accurately transcribing nutritional values.
- CB-OCR-visionOCR-3B (Model 4) successfully captures most content but introduces minor distortions into the spatial layout and alignment of tabular borders.

This example emphasizes that while all CB-OCR models are capable of text extraction under tabular constraints, CB-OCR-Nanonets and CB-OCR-RolmOCR achieve the best trade-off between structure preservation and recognition accuracy.

Such in-depth visual analyses corroborate and strengthen the quantitative results reported in Table 3. Among the different models, CB-OCR-Qwen2-VL tends to perform more uniformly through detailed descriptive text content, such as LRCB and HRCB coffee product labels. However, CB-OCR-Nanonets and CB-OCR-RolmOCR are significantly more effective in dealing with relatively structured and even tabular layout formats, as can be seen from the input examples of the POIE dataset. The variations observed in the performance underscore the importance of using suitable OCR methods that are robust to various features and structures in target label types.

### 4.4. CB-OCR Models Detection Time Performance Analysis

In addition to accuracy, computation efficiency is another important factor when using CB-OCR in practice, particularly in real-time or resource-limited conditions. In order to compare the proposed CB-OCR models, we also performed a thorough analysis of inference time. This research will evaluate their computational performance properties and determine the best trade-off between recognition accuracy and processing speed.

**Figure 6:**
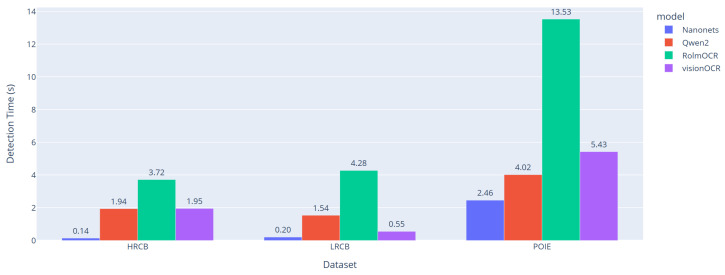
Average detection time of four proposed OCR methods on three datasets: HRCB, LRCB, and POIE.

In this work, inference time evaluation was consistently conducted using optimized image inputs under the same hardware and environment settings for fair comparison tests. All timing results are the average of several experiments accounting for system variability, and their results are summarized in Figure 6.

As shown in Figure 6, the experimental results demonstrate that various models and datasets perform differently, both in terms of computational efficiency. For the HRCB dataset, CB-OCR-Nanonets performed best for inference time at 0.14 s per image, leading to it retaining a significant computational edge against competing methods. CB-OCR-RolmOCR takes the longest time for processing, with a latency of 3.72 s. CB-OCR-Qwen2-VL and CB-OCR-visionOCR-3B achieve an intermediate performance with processing times of 1.94 and 1.95 s.

When taking the LRCB dataset into consideration, we also noted that CB-OCR-Nanonets had better computational efficiency with a 0.20 s inference time, in comparison to CB-OCR-RolmOCR, which required 4.28 s for processing. CB-OCR-Qwen2-VL performed similarly at 1.54 s, while CB-OCR-visionOCR-3B was very efficient, with only 0.55 s, making it the second fastest model for this dataset.

On the more complex POIE dataset of structured nutrition labels, all models showed increased inference times compared to those for CPD, particularly because the input images were now based on rich (structured) content. However, even with the increased computation load presented by this larger image size, our CB-OCR-Nanonets approach still achieved efficient processing in 2.46 s. CB-OCR-Qwen2-VL performed comparably at 4.02 s, and CB-OCR-visionOCR-3B took 5.43 s of processing time. The CB-OCR-RolmOCR had the slowest processing speed of 13.53 s, highlighting that this model architecture carries a serious computational cost for structured document analysis.

These detailed timing analyses offer valuable insights into the computational trade-offs of various CB-OCR architectures. CB-OCR-Nanonets always needed fewer MOPs than the other approaches on all three datasets, proving to be the most efficient method and being preferable for real-time applications as well. CB-OCR-visionOCR-3B exhibits a robust variable performance, demonstrating strong efficiency on the LRCB dataset and a modest performance on the other datasets. Even though CB-OCR-RolmOCR may have advantages in certain accuracy scores, the additional amount of computation time is much higher for this model for all datasets.

The performance results demonstrate the necessity of model selection techniques that take both the recognition performance and computational cost into consideration. For fast throughput or in computationally low-end deployment environments, the speed performance benefit of CB-OCR-Nanonets is favorable. CB-OCR-Qwen2-VL provides a trade-off between processing speed and computational resources and could be well suited to use cases with moderate performance requirements.

This extensive study emphasizes the need for a more encompassing evaluation approach, which must not only consider performance in terms of accuracy statistics but also practical deployment aspects such as inference efficiency, resource consumption, and real-world scenario needs when choosing among OCR model variants optimized for specific use cases.

### 4.5. Performance Comparison with Baseline OCR Methods

To extensively investigate our proposed CB-OCR-RolmOCR model, we conducted comparative studies with baseline OCR methods on three standard datasets. The baseline methods are represented by DocTR, PaddleOCR, EasyOCR, and Tesseract, which are state-of-the-art general-purpose OCR technologies. The performance comparison at the baseline is shown in Table 4.

#### 4.5.1. Performance Analysis by Dataset

**LRCB Dataset Results:** We summarize the comparative results for various evaluation factors that confirm the superior performance of CB-OCR-RolmOCR in Table 4. In LRCB, CB-OCR-RolmOCR is highly competitive compared to all seven other metrics with strong baselines. CB-OCR-RolmOCR scores an Exact Match of 0.7852, which is 72.8% better than the second highest baseline (DocTR at 0.4544). Our Cosine similarity performance of 0.8099 is much better than the former result (EasyOCR: 0.6908) for low-resolution coffee bean packaging text recognition, which suggests that it has a better semantic understanding ability.

**Results on HRCB Dataset:** CB-OCR-RolmOCR is effective for high-resolution images despite the strong competition. For this dataset, we do not report the results using DocTR (indicated in Table 4) since the model failed to detect text labels in the HRCB dataset. This failure was due to the extreme resolution and complex layouts of several of the high-resolution coffee packaging images, which caused DocTR’s pre-trained text localization module to produce empty detections. No memory overflow occurred; the issue arises from the model’s difficulty handling highly cluttered, reflective, or curved surfaces. CB-OCR-RolmOCR achieves the best scores on all of the introduced measures, in particular the Jaccard index (0.3641), Exact Match (0.5280), set performance, and ROUGE scores. The ROUGE-1 score of 0.6495 significantly outperforms those of the classical OCR methods, with Tesseract achieving only 0.2332. These results demonstrate CB-OCR-RolmOCR’s superior textual and structural integrity preservation in high-resolution imaging conditions.

**POIE Dataset Results:** We evaluate our model on the POIE dataset to demonstrate the effectiveness of CB-OCR-RolmOCR in structured document analysis. CB-OCR-RolmOCR also scores highest for six out of the eight performance metrics, showing particularly strong results in ROUGE-1 (0.9136) and ROUGE-2 (0.8587). These scores are remarkably higher than those of the previous baselines, i.e., 0.8590 and 0.7209 for DocTR, respectively. However, for every evaluation metric, the best performance among the baselines varies: DocTR achieves the largest Levenshtein similarity (0.3808), and Tesseract has the highest Cosine similarity (0.7738), which shows that different baseline performs well for certain semantic information. The Exact Match performance of 0.7525 demonstrates that CB-OCR-RolmOCR can extract structured data such as nutritional facts and ingredient tables with high accuracy.

#### 4.5.2. Baseline Method Analysis

PaddleOCR achieves the lowest performance on all three datasets, particularly for semantic similarity metrics, indicating challenges in handling ambiguous product label recognition. In contrast, EasyOCR and Tesseract show a moderate performance, but both are outperformed by CB-OCR-RolmOCR.

The effectiveness of our proposed approach for the recognition of coffee bean product labels is confirmed by the better performance on various datasets and evaluation measures provided by CB-OCR-RolmOCR. The model performs well in logical reasoning, indicated by the high Cosine of similarity between two datasets. It also demonstrates structural preservation and achieves excellent ROUGE scores, suggesting the improvement in content organization and the great Exact Match of correctly extracted text.

These results emphasize that after domain-specific optimization and better preprocessing methods, OCR can optimize the performance substantially for specific applications. These results establish CB-OCR-RolmOCR as a significant improvement over the current baseline OCR methods for coffee product label recognition in terms of effectiveness, both by providing better accuracy, a better semantic understanding, and better text structural preservation, which is important for an automated product information extraction system.

#### 4.5.3. Detection Time Performance Analysis

**Figure 7:**
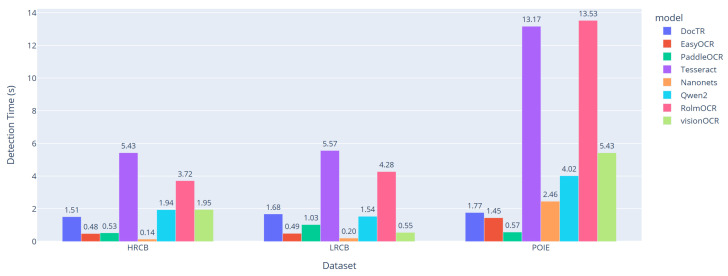
Average detection time comparing four proposed OCR methods with baseline OCR models across three datasets.

For a thorough investigation, we also compared the average detection time between four conventional OCR methods and our proposed approaches. The findings are illustrated in Figure 7.

As expected, the detection time depends greatly on the method and the dataset. For the HRCB dataset, the traditional OCR methods perform reasonably well, with average times of 0.14 s for PaddleOCR, 0.48 s for EasyOCR, and 1.51 s for DocTR. Tesseract, however, is considerably slower at 5.43 s. Among our methods, CB-OCR-Nanonets is the fastest at 1.94 s, followed closely by CB-OCR-visionOCR-3B (1.95 s) and CB-OCR-RolmOCR (3.72 s).

Performance shifts with the LRCB dataset. PaddleOCR still leads in speed at 0.20 s, followed by EasyOCR (0.49 s) and DocTR (1.68 s). Tesseract’s processing time rises to 5.57 s, reflecting its limitations on low-resolution images. Our methods show a varied performance: CB-OCR-visionOCR-3B leads at 0.55 s, while CB-OCR-RolmOCR takes 4.28 s. CB-OCR-Nanonets and CB-OCR-Qwen2-VL fall in between, with average times of 1.54 s and 1.03 s, respectively.

The POIE dataset is the most computationally intensive. Among the baseline methods, EasyOCR (1.45 s), CB-OCR-visionOCR-3B (0.57 s), and DocTR (1.77 s) remain practical, while Tesseract reaches 13.17 s. For our approaches, CB-OCR-RolmOCR is the slowest at 13.05 s, whereas CB-OCR-Nanonets (2.46 s), CB-OCR-Qwen2-VL (4.02 s), and CB-OCR-visionOCR-3B (5.43 s) achieve more moderate processing times.

This analysis highlights the trade-offs between accuracy and computational cost. While CB-OCR-RolmOCR generally offers higher accuracy (Table 4), it requires more processing time, especially on complex datasets like POIE. The increased processing time is due to enhanced preprocessing, domain-specific optimizations, and more sophisticated semantic understanding embedded in the model.

For near-real-time applications, high-speed methods such as PaddleOCR and EasyOCR are advantageous, offering efficiency at the modest expense of accuracy. In contrast, CB-OCR-RolmOCR prioritizes precision and holistic text interpretation, making it ideal for scenarios where the maximum accuracy is essential, such as detailed product information extraction, quality control, and automated inventory management.

Overall, these results emphasize the importance of selecting an OCR method that balances accuracy, average processing time, and real-time constraints based on the specific application.

## 5. Conclusions

This paper proposes an evaluation framework of advanced CB-OCR models developed exclusively for coffee bean product label recognition. It involves accuracy and computational effectiveness comparisons on three representative datasets, LRCB, HRCB, and POIE. We present extensive experimental verification evidencing that the proposed CB-OCR-RolmOCR model always outperforms for text recognition accuracy while successfully preserving semantic content integrity. It is especially good at handling descriptive and complicated label layouts, which are common in specialty coffee offerings.

Through our comparison, we find various fortes in different OCR models. Compared to the state-of-the-art methods on robustness in Table 1, CB-OCR-RolmOCR outperforms all of these baselines and significantly improves the performance with each evaluation metric. Also, we achieve high Exact Match scores of 0.7852 on the LRCB dataset (+72.8% higher than DocTR), 0.5280 on the HRCB dataset, and 0.7525 on the POIE dataset. These findings emphasize the power of domain-aware optimization and improved preprocessing techniques in dedicated OCR scenarios.

We provide a computational performance analysis and describe the trade-offs between accuracy and processing speed. With respect to the traditional baseline methods, namely DocTR, PaddleOCR, EasyOCR, and Tesseract, CB-OCR-RolmOCR requires a relatively longer analysis time, especially on the POIE dataset (with 13.53 s). In terms of high-accuracy applications (most, if not all, detailed property information extraction, quality control systems, automated inventory management), CB-OCR-RolmOCR offers better value. On the other hand, when real-time text extraction is necessary, conventional methods such as PaddleOCR (0.14–0.20 s) are faster yet less accurate.

The results highlight the importance of domain-specific OCR techniques for product label recognition pipelines. The performance, shown to be better than that of the traditional OCR methods, has proven that these advanced techniques can provide promising results for solving challenging consumer product package-handling-oriented natural scene text recognition problems. CB-OCR-RolmOCR’s superior semantic understanding (high Cosine similarity scores), structural preservation (superior ROUGE scores), and unsolved content matching a provide robust approach for a coffee product label recognition system.

In our future work, we will investigate the incorporation of automatic annotation to save human labor and more sophisticated preprocessing processes (such as image enhancement and noise deletion using special prior algorithms for package environments, as well as humanless label annotation efforts) to improve the recognition rate under difficult or extremely imaging conditions by incorporating lightweight semantic-aware or self-supervised dehazing modules [53]. The findings from this extensive study conclude that CB-OCR-RolmOCR is an important contribution to OCR in specialized domains, acting as a strong baseline system for automated product information extraction systems in the food and beverage industry, and possibly elsewhere.

## Figures and Tables

**Table 1:** Dataset statistics and characteristics.

Feature	LRCB	HRCB
Total Images	656	503
Default Resolution	640×480	2560×1920
Package Types	15	12
Lighting	5	5
Rotation Variants	4	4

**Table 2:** Hyperparameter setting of CB-OCR models.

Parameter	Value Setting
Precision	torch.float16 (half precision)
Unsharp mask	Radius = 2, Percent = 150, Threshold = 3
OCR Processor	AutoProcessor with chat template
Generation max tokens	1024
Temperature	0.1
Sampling method	Top-p sampling, p=0.9
Batch decoding	Skip special tokens, no cleanup of tokenization spaces
Confidence heuristic	Length of detected text; minimum length 5
Image formats	.png, .jpg, .jpeg, .bmp, .gif

**Table 3:** Performance comparison of our proposed OCR models across datasets. Best values per dataset are in **bold**.

Dataset	Model	Levenshtein	Jaccard	Cosine	Exact Match	BLEU	ROUGE-1	ROUGE-2	ROUGE-L
LRCB	CB-OCR-Nanonets	0.1577	0.0340	0.0295	0.0288	0.0031	0.0594	0.0283	0.0284
	**CB-OCR-RolmOCR**	**0.2486**	**0.4045**	**0.8099**	**0.7852**	**0.3322**	**0.7302**	**0.6027**	**0.1343**
	CB-OCR-visionOCR-3B	0.1853	0.0721	0.0968	0.0723	0.0141	0.1284	0.0684	0.0451
	CB-OCR-Qwen2-VL	0.2115	0.2947	0.7260	0.4016	0.2255	0.5120	0.3765	0.1191
HRCB	CB-OCR-Nanonets	0.1258	0.0266	0.0068	0.0096	0.0002	0.0307	0.0124	0.0201
	**CB-OCR-RolmOCR**	0.2117	**0.3641**	**0.3846**	**0.5280**	**0.3123**	**0.6495**	**0.5333**	**0.1556**
	CB-OCR-visionOCR-3B	0.2170	0.1997	0.2161	0.2271	0.0717	0.3651	0.2600	0.1086
	CB-OCR-Qwen2-VL	**0.2320**	0.2795	0.2272	0.2411	0.0952	0.3684	0.2719	0.1097
POIE	CB-OCR-Nanonets	0.1738	0.2107	0.4311	0.1410	0.0136	0.2703	0.2357	0.1929
	**CB-OCR-RolmOCR**	**0.3700**	**0.4148**	0.3084	**0.7525**	**0.3006**	**0.9136**	**0.8587**	**0.4047**
	CB-OCR-visionOCR-3B	0.2243	0.2877	0.5959	0.2175	0.0466	0.3879	0.3442	0.2343
	CB-OCR-Qwen2-VL	0.1855	0.1699	**0.6640**	0.1660	0.0121	0.3193	0.2541	0.2025

**Table 4:** Performance comparison between baseline OCR models and proposed CB-OCR-RolmOCR across three datasets. Best values per dataset and metric are in **bold**.

Dataset	Model	Levenshtein	Jaccard	Cosine	Exact Match	BLEU	ROUGE-1	ROUGE-2	ROUGE-L
LRCB	DocTR [17]	0.2298	0.1897	0.3310	0.4544	0.1625	0.4990	0.2865	0.1021
	PaddleOCR [18]	0.1531	0.0056	0.0151	0.0198	0.0000	0.0914	0.0089	0.0430
	EasyOCR [16]	0.2194	0.1129	0.6908	0.2197	0.0429	0.3212	0.1147	0.0733
	Tesseract [15]	0.2207	0.1152	0.2932	0.2858	0.0436	0.3045	0.1080	0.0679
	**CB-OCR-RolmOCR**	**0.2486**	**0.4045**	**0.8099**	**0.7852**	**0.3322**	**0.7302**	**0.6027**	**0.1343**
HRCB	DocTR [17]	—	—	—	—	—	—	—	—
	PaddleOCR [18]	0.1585	0.0042	0.0018	0.0028	0.0000	0.0750	0.0017	0.0325
	EasyOCR [16]	0.2217	0.0978	0.2091	0.1418	0.0172	0.2426	0.0906	0.0767
	Tesseract [15]	0.2379	0.1046	0.1367	0.1779	0.0447	0.2332	0.0871	0.0713
	**CB-OCR-RolmOCR**	**0.2117**	**0.3641**	**0.3846**	**0.5280**	**0.3123**	**0.6495**	**0.5333**	**0.1556**
POIE	DocTR [17]	**0.3808**	0.3463	0.7602	0.6927	0.3546	0.8590	0.7209	0.3748
	PaddleOCR [18]	0.1129	0.0051	0.0015	0.0011	0.0000	0.0627	0.0027	0.0474
	EasyOCR [16]	0.3407	0.1599	0.7484	0.4096	0.1100	0.5640	0.2875	0.2594
	Tesseract [15]	0.3344	0.1934	**0.7738**	0.5126	0.1917	0.6674	0.4210	0.2969
	**CB-OCR-RolmOCR**	0.3700	**0.4148**	0.3084	**0.7525**	**0.3006**	**0.9136**	**0.8587**	**0.4047**

## Data Availability

https://huggingface.co/datasets/Thi-Thu-Huong/Coffee-Bean-Package-Images-OCR (accessed on 5 October 2025).

## Abbreviations

The following abbreviations are used in this manuscript:

CLAHE	Contrast Limited Adaptive Histogram Equalization
DB	Differentiable Binarization
DocTR	Document Text Recognition
OCR	Optical Character Recognition
IoU	Intersection over Union
LRCB	Low-Resolution Coffee Bean
LCS	Longest Common Subsequence
VL	Vision–Language
VLM	Vision–Language Model
